# Controllable Spin Switching in a Single-Molecule Magnetic Tunneling Junction

**DOI:** 10.1186/s11671-021-03531-0

**Published:** 2021-05-01

**Authors:** Zhengzhong Zhang, Ya Wang, Haiou Wang, Hao Liu, Liming Dong

**Affiliations:** 1 Faculty of Mathematics and Physics, Huaiyin Institute of Technology, Huaian, China; 2 School of mechanical engineering and information, Shanghai Urban Construction Vocational College, Shanghai, China; 3 Institute of Materials Physics, Hangzhou Dianzi University, Hangzhou, China; 4 School of Automotive Engineering, Changshu Institute of Technology, Changshu, China

**Keywords:** Molecular spintronics, Single-molecule magnet, Spin-polarized current

## Abstract

A new type of spin-current filter is proposed that consists of a single-molecule magnet (SMM) coupled to two normal metal electrodes. It is shown that this tunneling junction can generate a highly spin-polarized current, whose spin polarization can be switched by means of magnetic fields and gate voltages applied to the SMM. This spin switching in the SMM tunnel junction arises from spin-selective single-electron resonant tunneling via the lowest unoccupied molecular orbit of the SMM. The electron current spectrum is still spin polarized in the absence of an external magnetic field, which can help to judge whether the molecule’s spin state has reached the ground-state doublet $|\pm S\rangle$. This device can be realized with current technologies and may have practical use in spintronics and quantum information.

## Introduction

With the development of materials science, nanoscale molecular electronic devices have been extensively studied in recent years with regard to their potential applications in nanoscale devices and spintronics [1–3]. Due to their small size and low power consumption, many basic devices utilizing molecules have been demonstrated, including tunnel junctions with negative differential resistance [4], rectifiers [5], amplifiers [6] and data storage [7]. Unlike conventional semiconductor devices, molecular devices consisting of single molecules seem to be quite suitable for functioning as controllable molecular switches [8]. Although molecular-scale switching has been reported in atomic quantum point contacts [9–11], single-molecule junctions provide the additional flexibility of the ability to tune the on/off conductance states through molecular design. Following the successful measurement of current flows through individual molecules in the past few decades, various kinds of molecular switches have been reported, such as light-controlled molecular switches [12] and mechanically controlled single-molecule switches [13], which can be used to shift a device between high- and low-conductance states. However, all of these switching schemes enable only the adjustment of charge transport conductance, not spin-dependent transport features.

In recent years, a new type of molecular material known as a single-molecule magnet (SMM) has been demonstrated to be an appropriate candidate as a basic component of molecule-based spintronic devices [14]. In contrast to other molecules, an SMM is a molecule with a relatively large net spin moment (corresponding to the spin number *S*) and significant uniaxial magnetic anisotropy [15]. At low temperatures, an SMM will be trapped in one of two metastable spin states $|\pm S\rangle$ [16]. This bistability makes SMMs a suitable basis for memory cells [17, 18] and has motivated many efforts to investigate the other physical properties of SMMs. So far, the electron transitions between an SMM and normal metal [19–21] or superconductor [22] interfaces have been experimentally investigated, and the functionalities of writing and reading information to and from an SMM by means of magnetic fields and electric biasing have also been demonstrated in the $\hbox {TbPc}_{{2}}$ molecule [23]. Inspired by these works, it is expected that the spin polarization of the tunneling current in an SMM can also be switched by means of magnetic fields and gate voltages; however, no controllable switching schemes based on such an SMM tunneling junction have yet been proposed.

## Methods

**Fig. 1:**
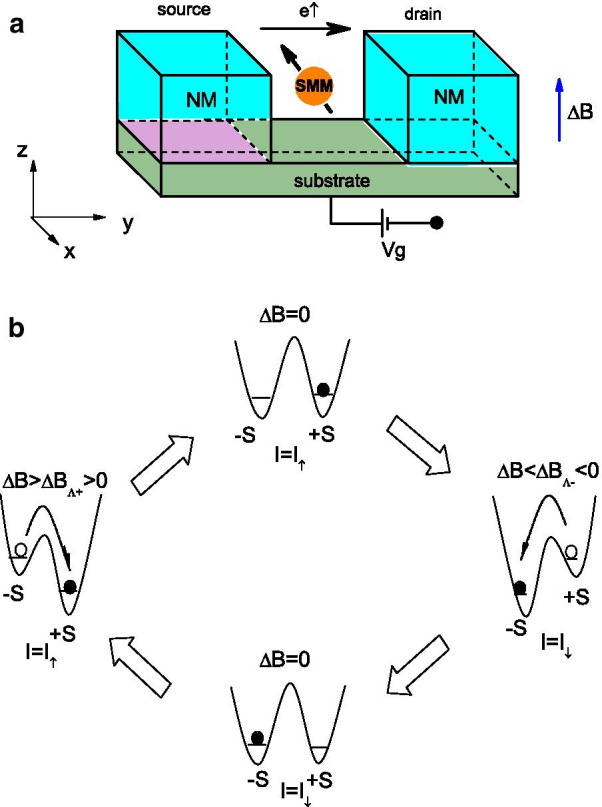
**a** Schematic diagram of a spin filter or spin memory consisting of an SMM coupled to a pair of nonmagnetic electrodes. **b** Schematic illustration of the switching of the SMM’s magnetization and the spin polarization of the tunneling current by means of external magnetic fields

In this letter, we present a new type of spin switching effect in an SMM tunnel junction that can be used to switch between pure spin-up and spin-down electronic currents by changing the external magnetic fields applied to the molecule. As shown in Fig. 1a, this nanostructure consists of an SMM connected to two normal metal electrodes. The energy level of the SMM is tuned by the gate voltage, and the spin-magnetization of the SMM can be switched by an external magnetic field. From Fig. 1b, we can see that the magnetic field controlled spin-injection in this device needs a two-step scheme: First, it applies a relatively larger external magnetic field to “write” a spin orientation of the SMM. The SMM’s core spin will be switched to one of two metastable spin states $\pm \,S$, depending on the magnetic field’s direction. And spin-injection process consists of using an electric bias exerted across the two leads in absence of a magnetic field. Due to the different chemical potential of the two leads and the magnetic anisotropy of the SMM, only electrons with the spin parallel to the SMM’s magnetization can flow through the junction [14], making the current highly polarized. The total Hamiltonian of the system is written as [24, 25]

1 $$\begin{aligned} H&= \varepsilon _{0}\sum _{\sigma }c_{\sigma }^{\dag }c_{\sigma }+Uc_{\uparrow }^{\dag }c_{\uparrow }c_{\downarrow }^{\dag }c_{\downarrow } -{\mathcal {D}}(S^{z})^{2}-J {\mathbf {s}}\cdot {\mathbf {S}} \nonumber \\&\quad -\Delta B(s^{z}+S^{z})+\sum _{k,\sigma ,\alpha }(t_{\alpha }a_{\alpha k\sigma }^{\dag }c_{\sigma }+t^{*}_{\alpha }c^{\dag }_{\sigma }a_{\alpha k\sigma }) \nonumber \\&\quad +\sum _{k,\sigma ,\alpha }\varepsilon _{k\sigma }a_{\alpha k\sigma }^{\dag }a_{\alpha k\sigma }. \end{aligned}$$

Here, $\varepsilon _{0}$ is the on-site energy of the lowest unoccupied molecular orbital (LUMO) of the SMM, which can be shifted by means of a gate voltage applied to the SMM; $c_{\sigma }^{\dag }$ ($c_{\sigma }$) is the electron creation (annihilation) operator with $\sigma$ as the Pauli spin index; *U* denotes the Coulomb repulsion energy; and ${\mathcal {D}}$ is the magnetic uniaxial anisotropy parameter. *J* is the exchange interaction between the spin of the conducting electrons, ${\mathbf {s}} =\sum \nolimits _{\sigma \sigma ^{\prime }}c_{\sigma }^{\dag }\sigma _{\sigma \sigma ^{\prime }}c_{\sigma ^{\prime }}/2$, at the LUMO level and the local spin ${\mathbf {S}}$. Since we assume that the easy axis of the molecule is the z-axis in the spin space, $\Delta B(s^{z}+S^{z})$ describes the Zeeman splitting associated with the magnetic field applied along this easy axis, where the ***g*** factor and the Bohr magneton $\mu _{B}$ are absorbed into $\Delta B$. $a_{\alpha k\sigma }^{\dag }$ ($a_{\alpha k\sigma }$) is the creation (annihilation) operator for electrons with momentum *k*, spin $\sigma$, and energy $\varepsilon _{k\sigma }$ in lead $\alpha$. The tunnel-coupling strength between the SMM and the normal metallic leads, which is denoted by $t_{\alpha }$, is independent of the momentum *k* and spin $\sigma$.

It is easy to diagonalize the Hamiltonian $H_{{\mathrm{mol}}}$ of the isolated SMM, i.e., the first five terms in Eq. (1). If we define ${\mathbf {S}}_{T}= {\mathbf {s}}+{\mathbf {S}}$, it can be shown that the eigenvalue *m* of $S_{T}^{z}$ is a good quantum number due to the commutation relation $[S_{T}^{z},H_{{\mathrm{mol}}}]=0$. In the following expressions, $|\bullet \rangle _{L({\mathrm{mol}})}$ represents the spin state of the LUMO (SMM). With $n=0,1,2$ defined as the number of electrons in the LUMO, the eigenenergies can be obtained as follows [26]: $\varepsilon _{|0,m\rangle }=-{\mathcal {D}}m^{2}-\Delta Bm$ for the eigenstates $|0,m\rangle =|0\rangle _{L}\otimes |m\rangle _{{\mathrm{mol}}}$, $\varepsilon _{|1,m\rangle ^{\pm }}=\varepsilon _0 -\Delta B m+J/4-{\mathcal {D}}(m^{2}+1/4)\pm \Delta \varepsilon (m)$ for the eigenstates $|1,m\rangle ^{\pm }=C_{1}^{\pm }|\downarrow \rangle _{L}\otimes |m+1/2\rangle _{{\mathrm{mol}}}+C_{2}^{\pm }|\uparrow \rangle _{L}\otimes |m-1/2\rangle _{{\mathrm{mol}}}$, and $\varepsilon _{|2,m\rangle }=2\varepsilon _0 +U-{\mathcal {D}}m^{2}-\Delta B m$ for the eigenstates $|2,m\rangle =|\uparrow \downarrow \rangle _{L}\otimes |m\rangle _{{\mathrm{mol}}}$. Here, $\Delta \varepsilon (m)=\sqrt{{\mathcal {D}}({\mathcal {D}}-J)m^{2}+(J/4)^{2}(2S+1)^{2}}$, and $C_{1}^{\pm }$ and $C_{2}^{\pm }$, which are given in Ref. [24], act as effective Clebsch–Gordan coefficients.

The transport process is dominated by sequential tunneling through the SMM level, while weak cotunneling and direct tunneling can be safely neglected. For the weak coupling between the SMM and leads, the master equation approach holds. The total spin-$\sigma$ current flowing through the SMM can be written as $I_{\sigma }=(I_{L\sigma }-I_{R\sigma })/2$, where $I_{L\sigma }$ ($I_{R\sigma }$) stands for the spin-$\sigma$ current flowing from the left (right) lead to the SMM, yielding

2 $$\begin{aligned} I_{\alpha \sigma }=-(e/h)\sum _{i,f}(n_{i}-n_{f})R_{\alpha \sigma }^{f\rightarrow i}P_{f}, \end{aligned}$$

such that the total current is equal to $I=\sum _{\sigma }(I_{L\sigma }-I_{R\sigma })/2$ and the spin polarization coefficient of the current is $\eta = \frac{ I_{\alpha \uparrow } - I_{\alpha \downarrow }}{ I_{\alpha }} \times 100\%$. In Eq. (2), $R_{\alpha \sigma }^{f\rightarrow i}$ denotes the transition rate between states $|i\rangle$ and $|f\rangle$, expressed as $R_{\alpha \sigma }^{f\rightarrow i}=\Gamma _{\alpha \sigma }[f(\varepsilon _{i}-\varepsilon _{f}-\mu _{\alpha })\langle i|c_{\sigma }^{\dag }|f\rangle ^{2}+f(\varepsilon _{i}-\varepsilon _{f}+\mu _{\alpha })\langle f|c_{\sigma }^{\dag }|i\rangle ^{2}]$, where $\Gamma _{\alpha \sigma }=2\pi D_{\alpha \sigma }|t_{\alpha }|^{2}$ is the line-width function for lead $\alpha$, with $D_{\alpha \sigma }$ being the density of states at $E_{F}$, and $f_{\alpha }$ is the Fermi function of lead $\alpha$ at temperature $T_{\alpha }$ and chemical potential $\mu _{\alpha }$. $P_{i}$ denotes the probability of finding the SMM in state $|i\rangle$. Following the numerical method suggested by Timm [26] and Shen [27], the time dependence of the probability $P_{i(t)}$ and the steady-state probability $P_{i(t\rightarrow \infty )}$ can be obtained by solving a set of rate equations ${\mathrm{d}}P_{i}/{\mathrm{d}}t=\sum _{f}R_{i,f}P_{i}$.

Here, numerical calculations are performed for $\hbox {Mn}_{{12}}$-Ac molecular tunnel junctions [19, 28], with spin number $S=10$, ${\mathcal {D}}=0.06$ meV, $J=0.1$ meV, and $U=25$ meV. The electrodes under consideration are made from normal metal, so the line-width functions are independent of the spin, i.e., $\Gamma _{\alpha \sigma }=\Gamma _{0}$ for simplicity.

**Fig. 2:**
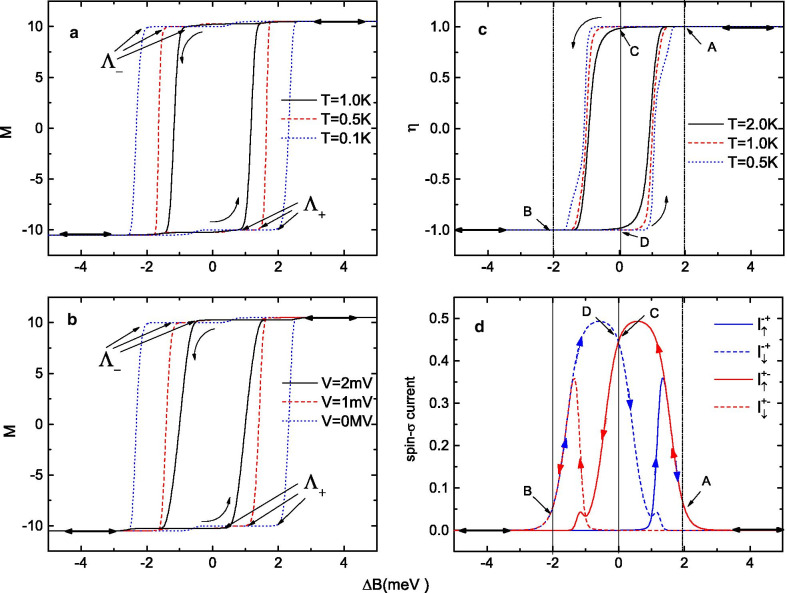
**a**, **b** Magnetic hysteresis loops of the SMM for **a** different equilibrium temperatures and **b** different bias voltages when the external magnetic field $\Delta B$ is scanned back and forth. **c** Spin polarization of the tunneling current for different equilibrium temperatures and **d** spin-$\sigma$ currents (scaled by $e\Gamma_{0} /\hbar$) at $T=0.5$ K when the external magnetic field $\Delta B$ is scanned back and forth under a fixed bias of $V=1$ mV

**Fig. 3:**
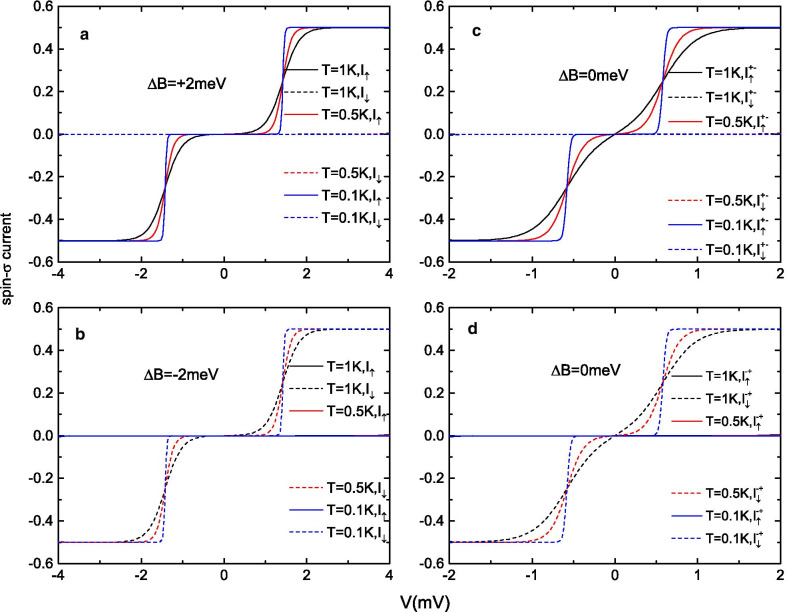
Spin-$\sigma$ currents $I_{\uparrow (\downarrow )}$ (scaled by $e\Gamma_{0} /\hbar$) (**a**, **b**) in the presence of external magnetic fields of **a** $\Delta B=+2$ meV, **b** $\Delta B=-\,2$ meV, **c**, **d** in the absence of a magnetic field as functions of the bias voltage

**Fig. 4:**
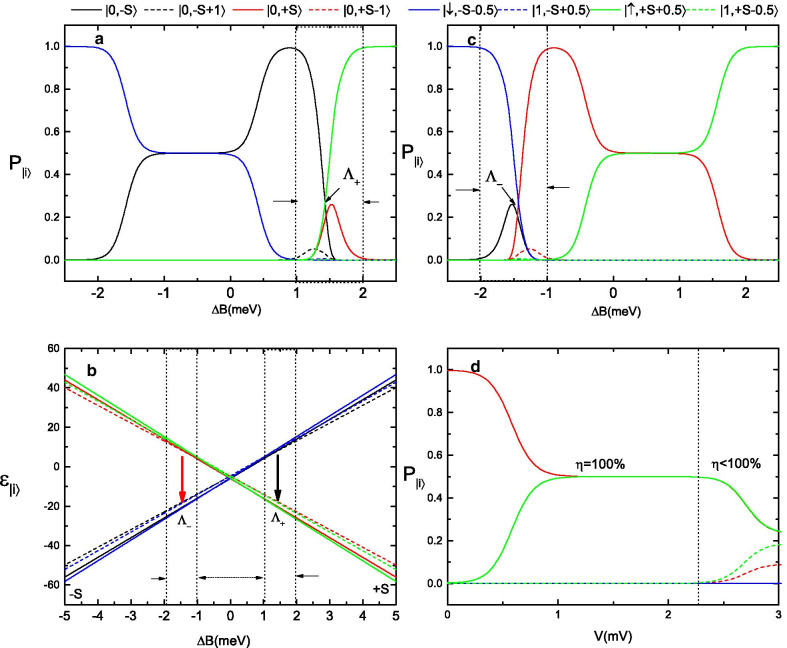
**a**, **c** Variations in the molecular state probabilities **a** as $\Delta B$ is scanned from ${-}\,5$ meV to ${+}\,5$ meV and **c** as $\Delta B$ is scanned from $+\,5$ meV to ${-}\,5$ meV. **b** Zeeman diagram for these spin states as $\Delta B$ changes from ${-}$ 5 meV to ${+}$ 5 meV. **d** Variations in the molecular state probabilities as functions of the bias voltage when the molecule’s spin state is initially prepared such that $P_{|0,+S\rangle }=1$ and $P_{i}=0$

**Fig. 5:**
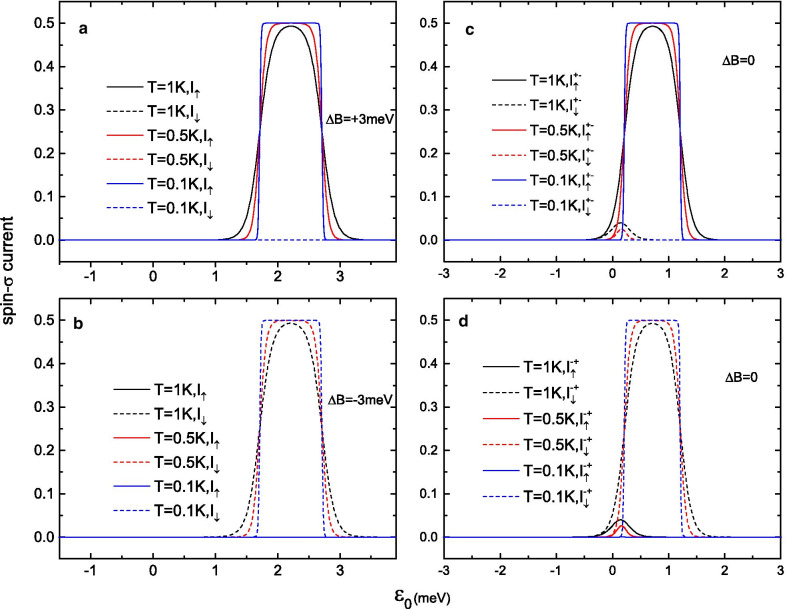
Spin-$\sigma$ currents $I_{\uparrow (\downarrow )}$ **a**, **b** in the presence of an external magnetic field of **a** $B=+2$ meV or **b** $B=-\,2$ meV and **c**, **d** in the absence of a magnetic field as functions of the molecular level $\varepsilon _{0}$

## Results and Discussion

First, we demonstrate how to use a magnetic field $\Delta B$ to “write in” the spin states of an SMM. In Fig. 2, we plot the magnetization of the SMM, the spin polarization $\eta$ of the current and the spin-$\sigma$ currents as functions of $\Delta B$, with a bias voltage exerted across the junction. Arrows indicate the scanning direction of the magnetic field, and the scanning process is assumed to be slow enough to allow the system to relax to a steady state. In Fig. 2a–c, it is shown that both the magnetization of the molecule and the spin polarization of the current exhibit loop structures when the magnetic field $\Delta B$ is scanned back and forth. For ease of description, we use $\Lambda _{-}$ to denote the reversal point when the magnetization of the SMM switches from $+S\rightarrow -S$ and $\Lambda _{+}$ to denote the reversal point for $-S\rightarrow +S$. The magnetization of the SMM is plotted as a function of $\Delta B$ for different equilibrium temperatures and bias voltages in Fig. 2a, b. It is evident that thermal fluctuations and electrical bias are both able to activate the magnetic reversal before $\Delta B$ exactly reaches the activation energy. Consequently, the magnetic hysteresis loop shrinks as the equilibrium temperature or bias voltage increases, and the distance between $\Lambda _{+}$ and $\Lambda _{-}$ decreases. However, no matter how much the magnetic hysteresis loop shrinks, the spin polarization coefficient of the tunneling current can always reach extremely high values of $\eta =\pm 100\%$ except when $\Delta B$ is near the two reversal points, $\Lambda _{+}$ and $\Lambda _{-}$. Furthermore, it is found that the spin polarization of the current in the small-magnetic-field regime $\Delta B_{\Lambda _{-}}< \Delta B<\Delta B_{\Lambda _{+}}$ is quite different from that in the large-magnetic-field regime $\Delta B<<\Delta B_{\Lambda _{-}}$ or $\Delta B>>\Delta B_{\Lambda _{+}}$. As shown in Fig. 2c, in the large-magnetic-field regime, the spin polarization coefficient $\eta$ of the tunneling current can be summarized as 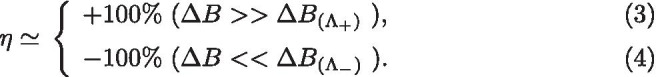


In this regime, for example, at point A (point B) in Fig. 2c, d, a given external magnetic field $\Delta B$ corresponds to a single, deterministic magnetization of the molecule, and only a $100\%$ spin-up (spin-down) electron current can flow through the junction. However, in the low-magnetic-field regime $\Delta B_{\Lambda _{-}}< \Delta B<\Delta B_{\Lambda _{+}}$, the original magnetization of the SMM can remain unchanged, and both the $+S$ and $-S$ spin directions can be retained. In Fig. 2d, we plot the $I_{\sigma }$-$\Delta B$ curves for the SMM junction at a fixed equilibrium temperature of $T=1$ K and a voltage of $V=1$ mV. It is clearly shown that a single given $\Delta B$ corresponds to two possible magnetizations of the molecule. If we use $I_{\sigma }^{+-}$ to denote the spin-$\sigma$ current when $\Delta B$ is scanned from $+5$ meV to $-5$ meV and $I_{\sigma }^{-+}$ to denote the current when the magnetic field is scanned in the opposite direction ($\Delta B$ changes from -5 meV to +5 meV), then both spin directions of the SMM at $+S$ or $-S$ can be read out with different spin polarization characteristics in the low-$\Delta B$ regime (such as at points C and D in Fig. 2c, d). In Fig. 2c, the spin polarization coefficient $\eta$ of the tunneling current in the small-magnetic-field regime $\Delta B_{\Lambda _{-}}< \Delta B<\Delta B_{\Lambda _{+}}$ can be summarized as
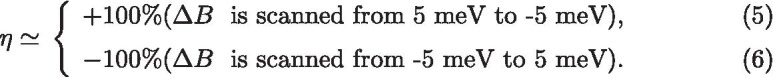


More importantly, as shown in Fig. 2d, we note that the tunneling current intensity at $\Delta B=0$, i.e., at point C or D, is much larger than that in the large-magnetic-field regime under the same bias voltage of $V=1$ mV. This means that this device will more easily generate spin-polarized electron currents in the absence of an external magnetic field, making it suitable as a spin filter or spin memory device.

To discuss the spin injection capabilities of this molecular junction, we plot the spin-$\sigma$ currents as functions of the bias voltage at a constant gate voltage and lower temperatures. Figure 3a, b shows the $I_{\uparrow (\downarrow )}$-*V* curves at large magnetic field values of $\Delta B= \pm 2$ meV (corresponding to the magnetic fields at points A and B in Fig. 2), while Fig. 3c, d shows the curves in the absence of $\Delta B$ (corresponding to points C and D in Fig. 2). No matter which magnetic field regime is chosen, the spin filtering feature is evident. As shown in Fig. 3a (Fig. 3b), only spin-up (spin-down) electrons can flow through the junction, while the electron current with the other spin direction is totally suppressed to zero by the spin selectivity of the SMM in the $+S$ ($-S$) direction. Similar results are obtained in Fig. 3c, d when the magnetic field $\Delta B$ is reduced to zero from the $+S$ and $-S$ directions. In the absence of $\Delta B$, the SMM must be trapped in one of the two bistable ground states $M=\pm S$. For this reason, both the $+S$ and $-S$ spin directions of the SMM can be well preserved in the $\Delta B=0$ regime. For example, if we scan $\Delta B$ from $+5$ meV to zero, $M=+S$ is saved, and a fully polarized spin-up current is obtained (see Fig. 3c). Furthermore, when the bias voltage is increased, the electron current in the absence of an external magnetic field reaches a relatively high current plateau earlier than in the case of a large magnetic field. As demonstrated in Fig. 3b, d, although there are no spin-up currents in both the $\Delta B=0$ meV and $\Delta B=-\,2$ meV regimes, the $I_{\downarrow }$ currents in Fig. 3d can reach up to $0.5e \Gamma _{0}/\hbar$ at $V\approx 0.7$ mV, while to reach the same amount of current in Fig. 3c, a larger bias voltage of at least $V>1.5$ mV is needed.

To clarify the underlying physics in Figs. 2 and 3, we plot the molecular state probabilities $P_{|0,\pm S\rangle }$, $P_{|0, S-1\rangle }$, $P_{|0, -S+1\rangle }$, $P_{|\uparrow , S+1/2\rangle }$, $P_{|\downarrow , -S-1/2\rangle }$, $P_{|1, S-1/2\rangle }$ and $P_{|1, -S+1/2\rangle }$ as functions of $\Delta B$ when the magnetic field is scanned back and forth at a fixed equilibrium temperature of $T=0.5$ K and a bias voltage of $V=1$ mV. In Fig. 4a, $\Delta B$ is scanned from $-5$ meV to $+5$ meV slowly enough to allow the system to relax to the steady state. It is shown that in the large magnetic field regime $\Delta B<-2$ meV, all states’ probabilities are equal to zero except $P_{|\downarrow , -S-1/2\rangle }=1$, which means that the SMM’s spin state is fixed in the $-S$ direction and one spin-down electron is trapped in the molecule’s LUMO level by the external magnetic field. For a relatively large value of the Coulomb repulsion energy ($U=25$ meV) and a spin-down electron trapped in the LUMO level, a spin-up electron cannot exist at the SMM’s level, and the electron current is blocked. When $\Delta B$ increases from $-2$ meV to 1 meV, a nonzero molecular state probability $P_{|0,-S\rangle }$ emerges, and the electron current is dominated by the $\varepsilon _{|0,-S\rangle }\leftrightarrow \varepsilon _{|\downarrow , -S-1/2\rangle }$ transition. In this $\Delta B$ window, the SMM’s spin states can still be saved in the $-S$ direction, but spin-down electrons can tunnel through the SMM, resulting in a pure spin-down polarized electron current. However, when $\Delta B$ is further increased to the range of $[1\,{\text {meV}}, 2\,{\text {meV}}]$, the inelastic tunneling processes that lead to magnetic switching of the molecule’s spin take place. In this regime, nearly all the spin states of the SMM have a chance to be occupied, and the probabilities of two special states, $P_{|0,-S\rangle }$ and $P_{|\uparrow , +S+1/2\rangle }$, are much larger than those of any other states. More interestingly, the point where $P_{|0,-S\rangle }= P_{|\uparrow , +S+1/2\rangle }$ exactly corresponds to the point $\Lambda _{+}$ in Fig. 2a, indicating that the molecule’s magnetization is starting to reverse from $-S$ to $+S$. As $\Delta B$ continues to increase above 2 meV, all states’ probabilities decrease to zero except $P_{|\uparrow , S+1/2\rangle }\rightarrow 1$, which implies that the SMM’s spin state is fixed in the $+S$ direction and that the tunneling current will be switched “off” by one spin-up electron blocking the molecule’s LUMO level. On the other hand, if the magnetic field is scanned from $+5$ meV to $-5$ meV (see Fig. 4c), a similar process will occur again, and the reversal point $\Lambda _{-}$ corresponds to the point where $P_{|0,+S\rangle }= P_{|\downarrow , -S-1/2\rangle }$. In Fig. 4b, we present the Zeeman diagram for these spin states. Due to the large magnetic anisotropy of the SMM, the ground-state doublet with quantum numbers $M =\pm S$ ($S=10$ for $\hbox {Mn}_{{12}}$-Ac) is well separated from the excited states by an energy barrier of $DS^{2}_{z}\approx 60$ K. Moreover, the magnetic switching point $\Lambda _{(+)-}$ in Fig. 4 is approximately equal to 1.3 meV, which is close to the reversal point $2S|{\mathcal {D}}|$ in single magnetic atoms. In Fig. 4d, we plot the molecular state probabilities as functions of the bias voltage for a fixed temperature of $T=0.5$ K and a magnetic field of $\Delta B=0$. If we assume that the SMM is trapped in the $+S$ spin direction, then the electron tunneling process in Fig. 4d can be divided into two parts: (i) In the small-bias regime $V<2.5$ mV, the electron current is dominated by the $\varepsilon _{|0,+S\rangle }\leftrightarrow \varepsilon _{|\uparrow , S+1/2\rangle }$ transition, and only spin-up electrons can tunnel through the junction. (ii) When the bias voltage increases to the large-bias regime $V>2.5$ mV, although the bias is not large enough to overcome the energy barrier between the spin directions $+S$ and $-S$, spin states with higher energy in the $+S$ direction, such as $\varepsilon _{|0,+S-1\rangle }$ and $\varepsilon _{|1,+S-1/2\rangle }$, can be occupied, which will introduce additional extra channels for spin-down electron tunneling through the SMM. As a result, as the bias voltage continues to increase, the tunneling current will continue to grow, but the spin polarization coefficient $\eta$ will decrease.

Finally, the results for the spin-up current $I_{\uparrow }$ and spin-down current $I_{\downarrow }$ as functions of the gate voltage (on-site energy of the LUMO level $\varepsilon _{0}$) are calculated, with and without an external magnetic field (see Fig. 5). Under low temperatures, 100$\%$ spin-polarized electronic currents can be switched “on/off” by means of different gate voltage windows. When $\Delta B=\pm 2$ meV is applied, pure spin-$\sigma$ currents emerge in a certain gate voltage window of $0.8\,{\text {meV}}< \varepsilon _{0} < 2.8\,{\text {meV}}$, while $I_{\uparrow } = I_{\downarrow } = 0$ outside of this regime. As the equilibrium temperature *T* increases, the peaks of $I_{\sigma }$ become lower and broaden, but the high spin-polarized current seen at low temperatures is still maintained (see Fig. 5a, b). Unlike in the large-magnetic-field regime, the spin-$\sigma$ currents are switched “on” without an external magnetic field in the gate voltage window of $-0.8\,{\text {meV}}< \varepsilon _{0} <1.8\,{\text {meV}}$, and the spin polarization exhibits two different results (see Fig. 5c, d). In the gate voltage window of $0.8\,{\text {meV}}< \varepsilon _{0} <1.8\,{\text {meV}}$, $\pm \,100\%$ spin-polarized electronic currents can be generated under a small bias of V$=1$ mV, corresponding to points C and D in Fig. 2c. However, in the gate voltage window of $-0.8\,{\text {meV}}< \varepsilon _{0} <0.8\,{\text {meV}}$, the energy gaps between the states $|0,\pm S \rangle$ and $|1,\pm S\mp 0.5 \rangle$ become very small, and more spin states with higher energy in the $+S$ (or $-S$) spin direction can be reached by means of the bias voltage; thus, both spin-up and spin-down electrons can tunnel through the SMM. Consequently, the total spin polarization $\eta$ of the electric current is reduced in this gate voltage regime.

## Conclusion

In summary, we have proposed a three-state switching effect with two “on” states for spin-up and spin-down current switching as well as a current “off” state. Such spin-polarized current switching can be realized in an SMM (e.g., $\hbox {Mn}_{{12}}$-Ac) tunnel junction and arises from spin-selective single-electron resonant tunneling via the LUMO of the SMM. This three-state switching behavior can be controlled by means of magnetic fields and gate voltages, without spin-orbit interactions or magnetic leads, and is a good candidate for spintronic devices such as spin filters or spin memories in future spintronic circuits.

## Data Availability

The datasets used during the current study are available from the corresponding author of this article.

## Abbreviations

SMM: Single-molecule magnet
LUMO: Lowest unoccupied molecular orbital
Mn_12_-Ac: [Mn_12_O_12_(CH_3_CO_2_)_15_(H_2_O)_4_]
TbPc_2_: [(C_32_H_16_N_8_)_2_Tb^III^] complex
